# Supplementary material for: Interpretable machine learning decodes soil microbiome’s response to drought stress
Source: Environ Microbiome. 2024 May 29;19:35. doi: 10.1186/s40793-024-00578-1 (PMC11138018; doi:10.1186/s40793-024-00578-1)
Supplement: Supplementary file 1 — Additional file 1: Figure S1. Weekly watering scheme of the Sorghum-Drought test dataset. Table S1. Feature table pruning between the datasets. Figure S2. Relative abundances per rank of the Grass-Drought dataset. Figure S3. Significant taxa intersections between DAA tools per rank of the Grass-Drought dataset. Figure S4. ROC curves per rank of the Grass-Drought dataset. Table S2. Logistic regression performance of the Grass-Drought dataset. Figures S5 and S6. Taxon enrichment, significance and importance by DAA tools and SHAP values of the Grass-Drought dataset. Table S3. Random forest classifier performance on the Grass-Drought dataset excluding the Hold-Out dataset. Table S4. Random forest classifier performance on the Hold-Out dataset of the Grass-Drought dataset. [file 40793_2024_578_MOESM1_ESM.pdf]

Interpretable Machine Learning Decodes Soil Microbiome's Response to Drought Stress

Supplementary Figures and Tables

Michelle Hagen[1], Rupashree Dass[1], Cathy Westhues[1],
Jochen Blom[2], Sebastian J Schultheiss[1], Sascha Patz[1]

[1] Computomics GmbH
Eisenbahnstraße 1
Tübingen, 72072
Baden-Württemberg, Germany

[2] Bioinformatics & Systems Biology
Justus Liebig University Gießen
Heinrich-Buff-Ring 58
Gießen, 35390
Hesse, Germany

This document includes the following supporting information:

- **Fig. S1:** Weekly Watering Scheme of the Sorghum-Drought Test Dataset
- **Tab. S1:** Feature Table Pruning between the Datasets
- **Fig. S2:** Relative Abundances per Rank of the Grass-Drought Dataset
- **Fig. S3:** Significant Taxa Intersections between DAA Tools per Rank of the Grass-Drought Dataset
- **Fig. S4:** ROC Curves per Rank of the Grass-Drought Dataset
- **Tab. S2:** Logistic Regression Performance of the Grass-Drought Dataset
- **Fig. S5 + S6:** Taxon Enrichment, Significance and Importance by DAA Tools and SHAP Values of the Grass-Drought Dataset
- **Tab. S3:** Random Forest Classifier Performance on the Grass-Drought Dataset Excluding the Hold-Out Dataset
- **Tab. S4:** Random Forest Classifier Performance on the Hold-Out Dataset of the Grass-Drought dataset

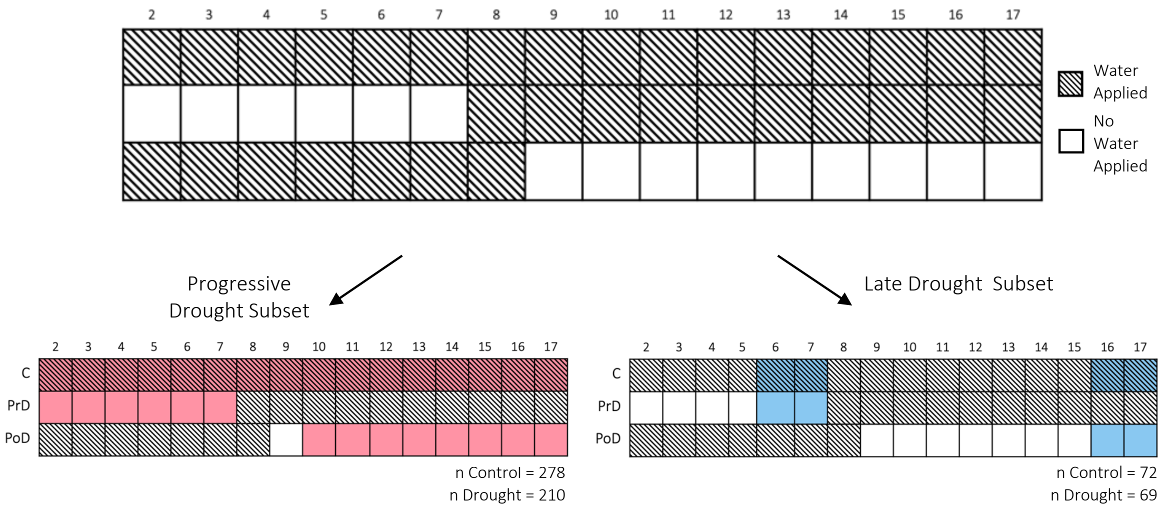

Fig. S1: Weekly Watering Scheme of the Sorghum-Drought Test Dataset. Watering scheme for the three groups 'Control' (C), 'Pre-Flowering Drought' (PrD), and 'Post-Flowering Drought' (PoD). Dashed lines indicate watering and white cells indicate no watering. The test dataset was subset into the 'Progressive Drought' subset (red) and 'Late Drought' subset (blue).

Tab. S1: Feature Table Pruning between the Datasets. Table displaying differences in the total number of different taxa between the Grass-Drought dataset as the dataset the RFC has been trained on and the Sorghum-Drought dataset as the test dataset. A high number of taxa added with zero counts in order to bring the feature tables of the test dataset into a suitable format for prediction also increases the level of potential sparsity.

Rank	Phylum	Class	Order	Family	Genus
Number of taxa Grass-Drought	26	60	131	186	330
Number of taxa sorghum-drought	33	69	148	186	311
Matching between both	25	54	111	148	198
Added with zero counts	1	6	20	38	132

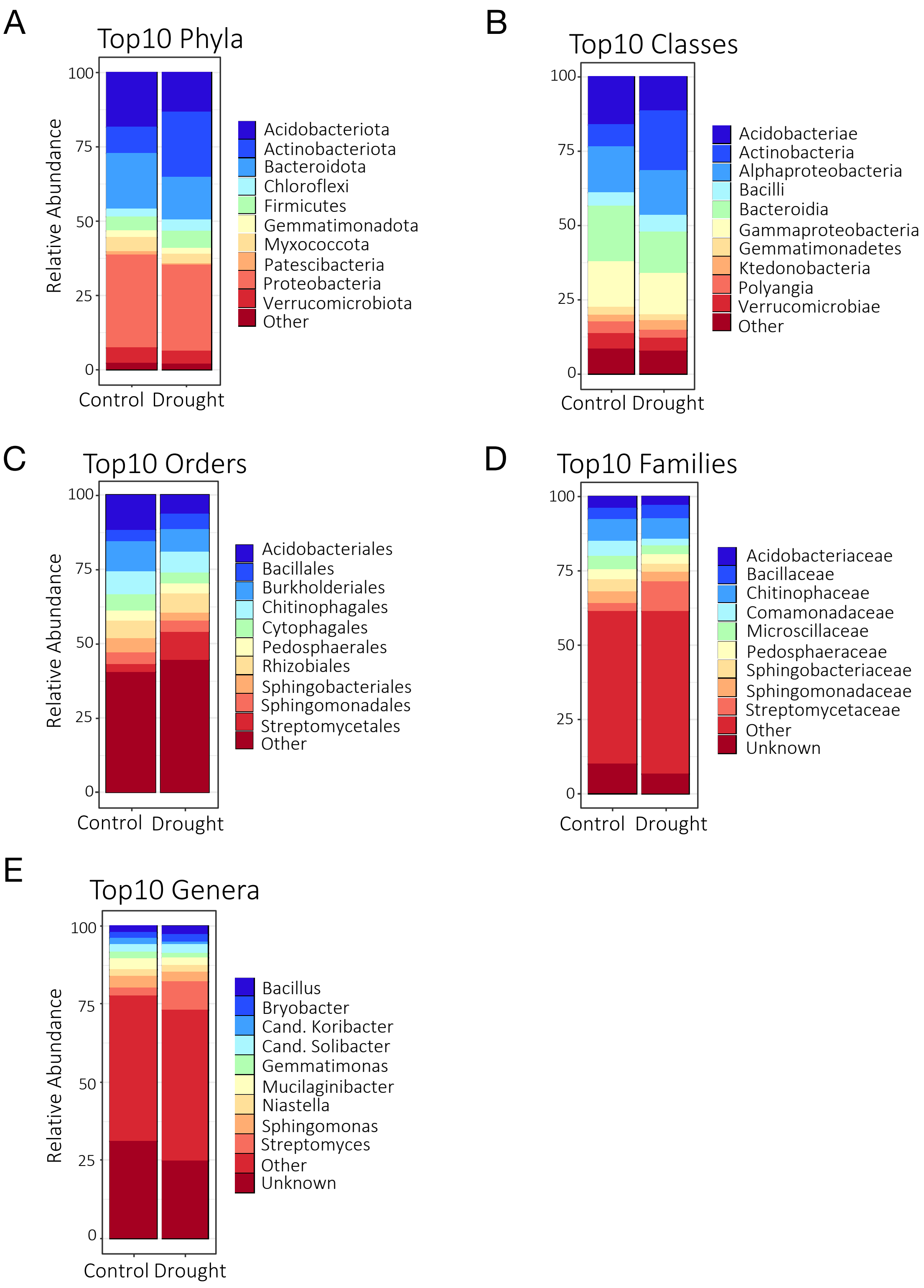

Fig. S2: Relative Abundances per Rank of the Grass-Drought Dataset. Bar plots displaying the relative abundance of the top 10 taxa between the 'Control' and 'Drought' groups on (A) Phylum, (B) Class, (C) Order, (D) Family, and (E) Genus level in alphabetical order.

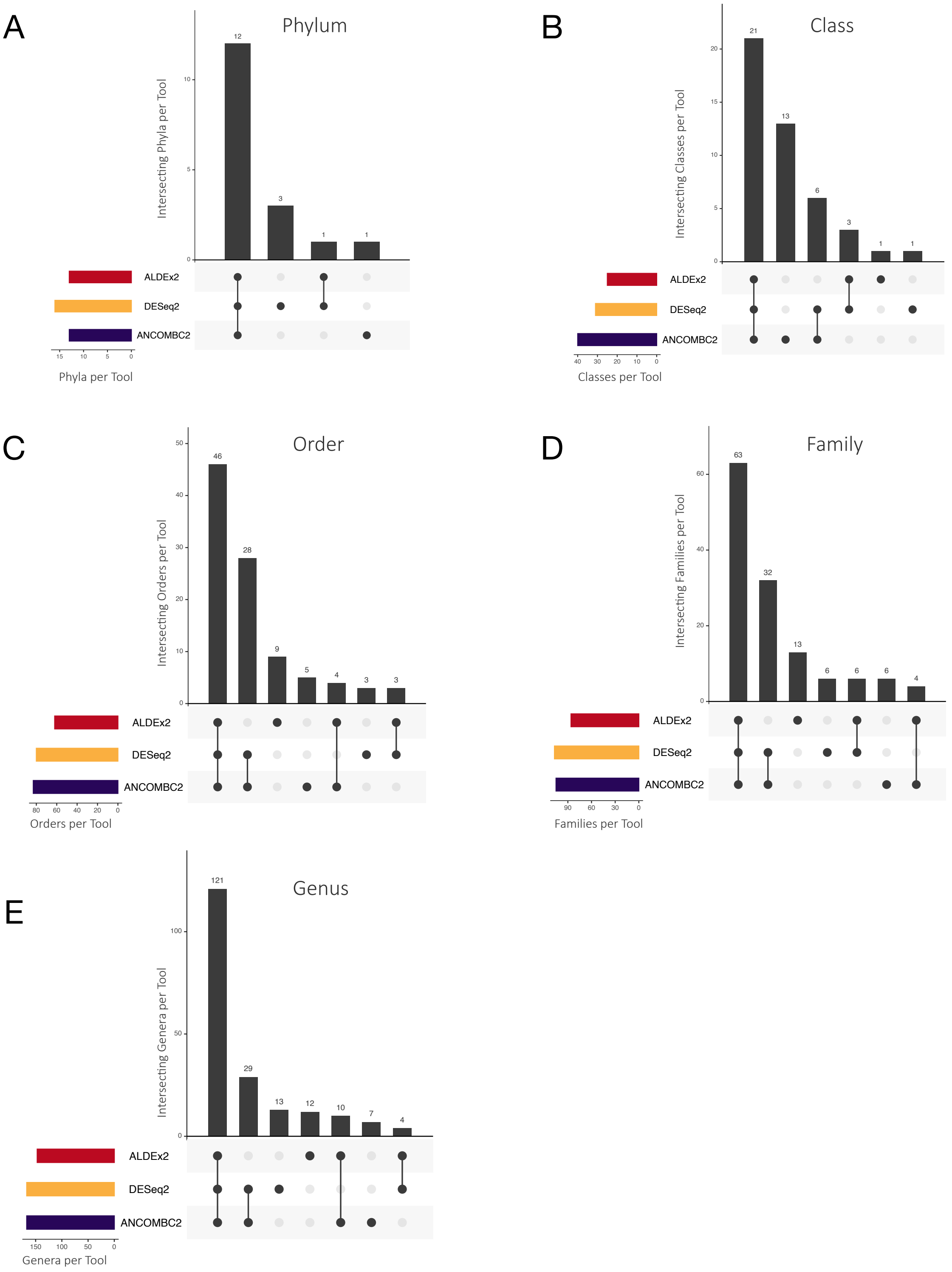

Fig. S3: Significant Taxa Intersections between DAA Tools per Rank of the Grass-Drought Dataset. Upset plots displaying the overlap and uniqueness of significant taxa identified by the three DAA methods 'ALDEx2', 'DESeq2', and 'ANCOM-BC2' on (A) Phylum, (B) Class, (C) Order, (D) Family, and (E) Genus level. The horizontal bars show the total number of taxa for each tool, while the vertical bars show the number of shared taxa between corresponding sets, sorted by the total number of shared taxa. All tools use an alpha threshold of 0.05 for significance.

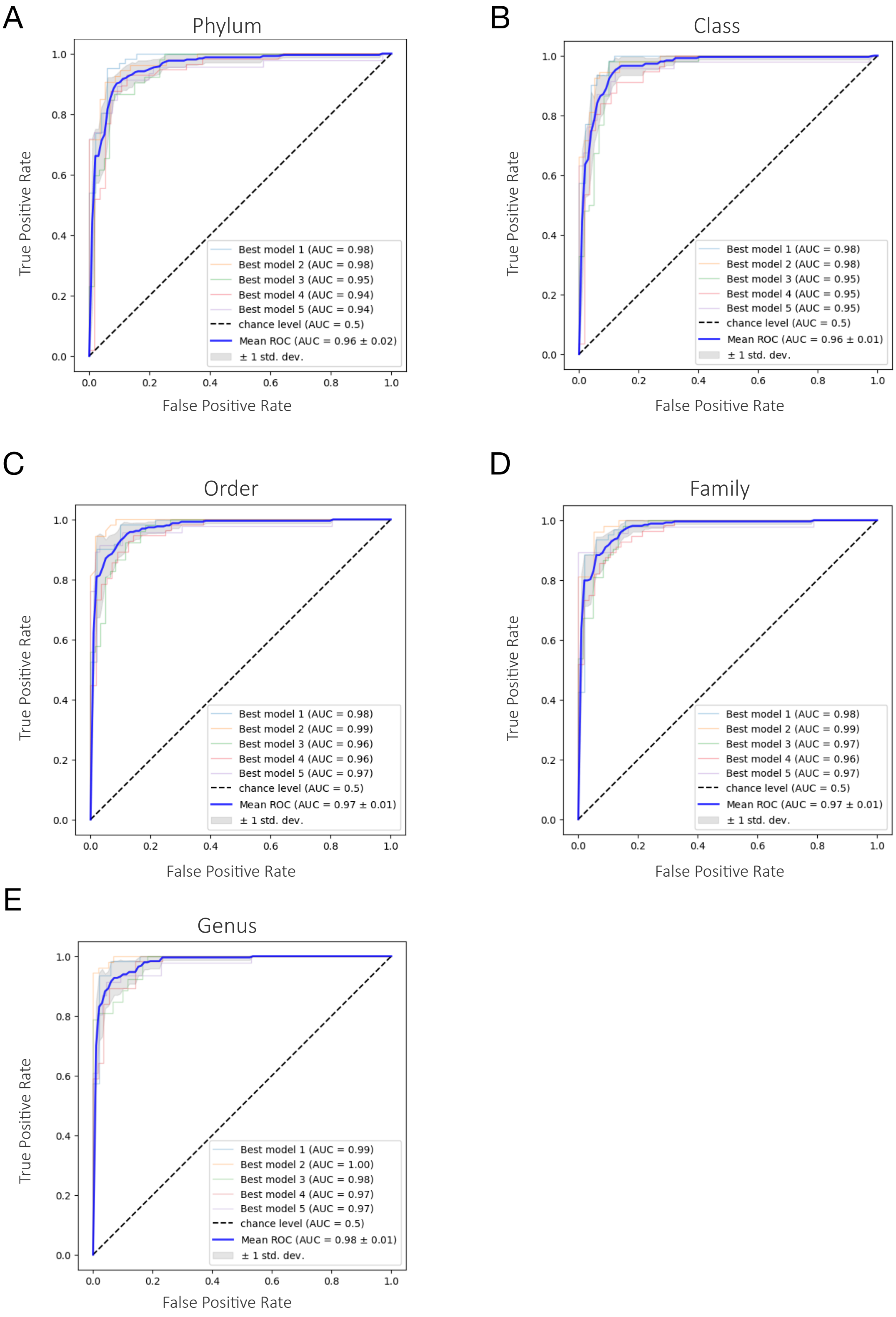

Fig. S4: ROC Curves per Rank of the Grass-Drought Dataset. Receiver Operating Characteristic (ROC) curve at (A) Phylum, (B) Class, (C) Order, (D) Family, and (E) Genus level, showing the area under the curve (AUC) for each fold of the nested cross-validation. The ROC curve displays the best model for each fold and the mean AUC.

In addition to the Random Forest Classifier, Logistic Regression was tested for the binary classification of drought stress. For this, a five-fold nested cross-validation was conducted. The Grass-Drought dataset was preprocessed in the same manner, and feature tables were created as in the same way as for the Random Forest Classifier. **Tab. S2** displays the performance across various taxonomic ranks.

Tab. S2: Logistic Regression Performance of the Grass-Drought Dataset. Table displaying the mean accuracy, F1 score, precision, recall, and AUC of the classifier on different taxonomic ranks of the Grass-Drought dataset, with the best-performing rank for each metric marked in bold. The Grass-Drought dataset underwent preprocessing, aligning with the approach utilized by the Random Forest Classifier, followed by a five-fold nested cross-validation.

**Metric**	Phylum	Class	Order	Family	Genus
Accuracy	0.836 ± 0.026	0.829 ± 0.028	0.900 ± 0.043	0.909 ± 0.035	**0.916 ± 0.028**
F1 score	0.821 ± 0.034	0.816 ± 0.040	0.897 ± 0.044	0.907 ± 0.033	**0.914 ± 0.030**
Precision	0.853 ± 0.066	0.831 ± 0.059	0.875 ± 0.050	0.887 ± 0.042	**0.889 ± 0.036**
Recall	0.796 ± 0.045	0.803 ± 0.044	0.920 ± 0.043	0.929 ± 0.028	**0.941 ± 0.036**
AUC	0.909 ± 0.030	0.914 ± 0.027	0.964 ± 0.019	0.964 ± 0.023	**0.968 ± 0.019**

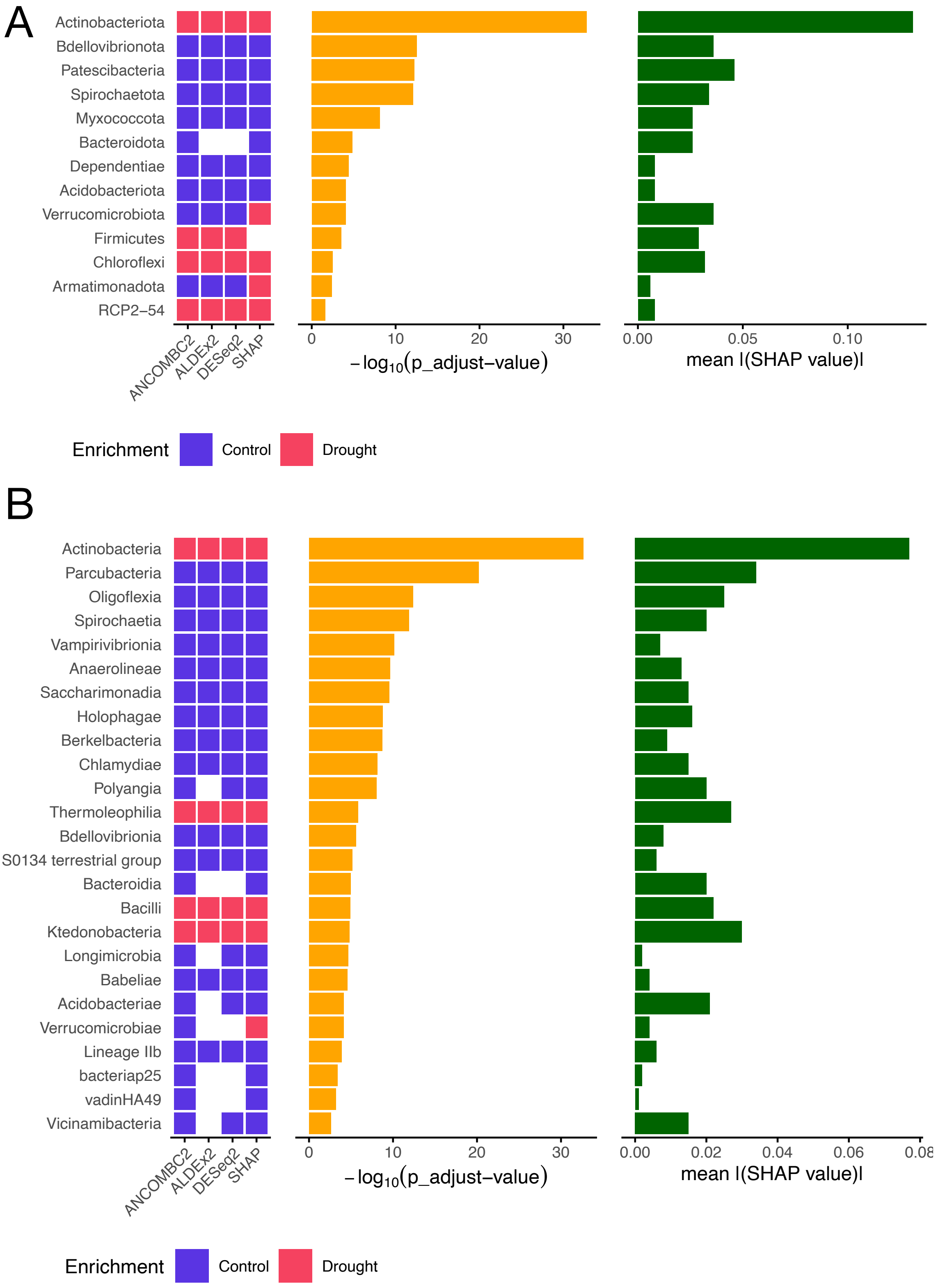

Fig. S5: Taxon Enrichment, Significance and Importance by DAA Tools and SHAP Values of the Grass-Drought Dataset. Binary heatmap showing the enrichment of the top significant taxa from ANCOM-BC2 on (A) Phylum and (B) Class level between 'Control' (blue) and 'Drought' (red) groups for the three methods used for DAA (DESeq2, ANCOM-BC2, ALDEx2) with an alpha <0.05, and SHAP values obtained from the RFC. Empty cells display no significant enrichment. Corresponding bar plots comparing -$\log_{10}$(p_adjust) values (orange) and mean(|SHAP value|) (green).

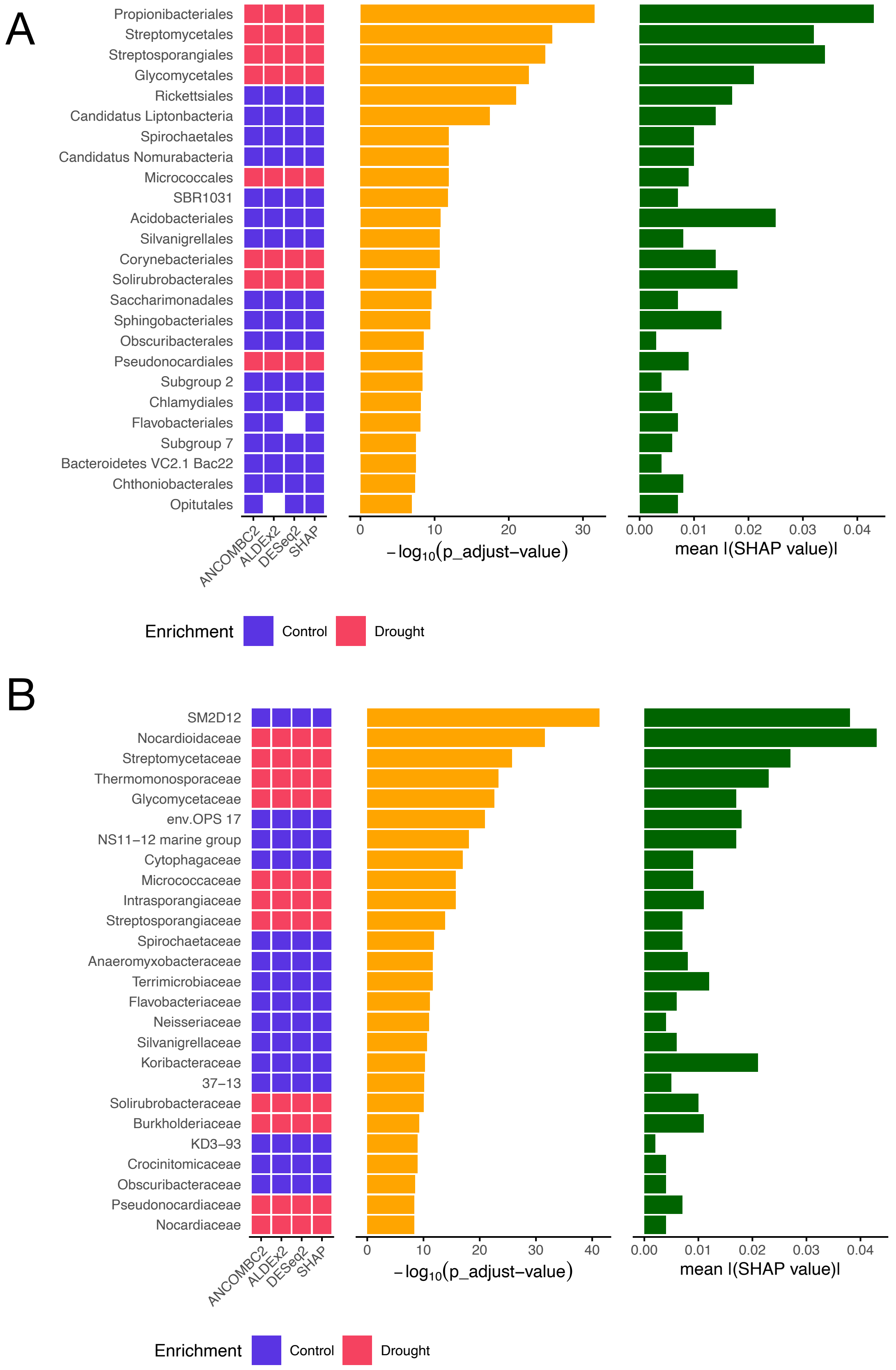

Fig. S6: Taxon Enrichment, Significance and Importance by DAA Tools and SHAP Values of the Grass-Drought Dataset. Binary heatmap showing the enrichment of the top significant taxa from ANCOM-BC2 on (A) Order and (B) Family level between 'Control' (blue) and 'Drought' (red) groups for the three methods used for DAA (DESeq2, ANCOM-BC2, ALDEx2) with an alpha <0.05, and SHAP values obtained from the RFC. Empty cells display no significant enrichment. Corresponding bar plots comparing -$\log_{10}$(p_adjust) values (orange) and mean(|SHAP value|) (green).

To test the robustness of the five-fold nested cross-validation, 20 % of the Grass-Drought dataset was set aside before any preprocessing steps to create an independent hold-out dataset while still preserving the ratio of the two watering regimes. Feature tables were constructed for the remaining samples of the Grass-Drought dataset (448 samples) and the hold-out dataset (112 samples). A five-fold nested cross-validation with hyperparameter tuning using a Random Forest Classifier was then conducted on the remaining Grass-Drought dataset. This bootstrapping process was performed five times using different, independently selected subsets. **Tab. S3** displays the mean performance of the five-fold nested cross-validation of all subsampling rounds, while **Tab. S4** showcases the classifier's performance throughout all subsampling rounds on an independent hold-out dataset, which the classifier has not been trained on.

Tab. S3: Random Forest Classifier Performance on the Grass-Drought Dataset Excluding the Hold-Out Dataset. Table displaying the average of the mean accuracy, F1 score, precision, recall, and AUC of the classifier on different taxonomic ranks of the reduced Grass-Drought dataset of five independent subsampling rounds, with the best-performing rank for each metric marked in bold.

**Metric**	Phylum	Class	Order	Family	Genus
Accuracy	0.882 ± 0.005	0.899 ± 0.006	0.901 ± 0.004	0.914 ± 0.005	**0.925 ± 0.004**
F1 score	0.875 ± 0.006	0.893 ± 0.007	0.897 ± 0.005	0.912 ± 0.004	**0.922 ± 0.004**
Precision	0.874 ± 0.009	0.889 ± 0.008	0.876 ± 0.006	0.891 ± 0.003	**0.902 ± 0.007**
Recall	0.879 ± 0.015	0.900 ± 0.019	0.922 ± 0.004	0.935 ± 0.010	**0.946 ± 0.006**
AUC	0.952 ± 0.004	0.957 ± 0.004	0.967 ± 0.004	0.968 ± 0.004	**0.975 ± 0.005**

Tab. S4: Random Forest Classifier Performance on the Hold-Out Dataset of the Grass-Drought dataset. Table displaying the average of the mean accuracy, F1 score, precision, recall, and AUC of the classifier on different taxonomic ranks of the Grass-Drought hold-out dataset of five independent subsampling rounds, with the best-performing rank for each metric marked in bold.

**Metric**	Phylum	Class	Order	Family	Genus
Accuracy	0.890 ± 0.034	0.909 ± 0.033	0.912 ± 0.028	0.927 ± 0.020	**0.939 ± 0.021**
F1 score	0.885 ± 0.037	0.909 ± 0.034	0.910 ± 0.029	0.926 ± 0.020	**0.930 ± 0.020**
Precision	0.891 ± 0.027	0.901 ± 0.033	0.899 ± 0.027	0.905 ± 0.026	**0.907 ± 0.025**
Recall	0.882 ± 0.050	0.915 ± 0.039	0.922 ± 0.033	0.949 ± 0.020	**0.954 ± 0.018**
AUC	0.958 ± 0.018	0.963 ± 0.017	0.970 ± 0.014	0.975 ± 0.012	**0.980 ± 0.012**
